# Lateral flow devices for samples collected by straw sampling method for postmortem canine rabies diagnosis

**DOI:** 10.1371/journal.pntd.0009891

**Published:** 2021-12-09

**Authors:** Milagros R. Mananggit, Daria L. Manalo, Nobuo Saito, Kazunori Kimitsuki, Alyssa Marie G. Garcia, Patricia Mae T. Lacanilao, Joely T. Ongtangco, Cornhlo R. Velasco, Maria Victoria A. del Rosario, Maria Glofezita O. Lagayan, Kentaro Yamada, Chun-Ho Park, Satoshi Inoue, Motoi Suzuki, Mariko Saito-Obata, Yasuhiko Kamiya, Catalino S. Demetria, Beatriz P. Quiambao, Akira Nishizono

**Affiliations:** 1 Regional Animal Disease Diagnostic Laboratory, Department of Agriculture Field Office III, San Fernando, Pampanga, Philippines; 2 Research Institute for Tropical Medicine, Muntinlupa City, Philippines; 3 Department of Microbiology, Faculty of Medicine, Oita University, Yufu, Japan; 4 School of Tropical Medicine & Global Health, Nagasaki University, Nagasaki, Japan; 5 Department of Agriculture, Bureau of Animal Industry, Quezon City, Philippines; 6 Laboratory of Veterinary Public Health, Department of Veterinary Medical Science, Faculty of Agriculture, University of Miyazaki, Miyazaki, Japan; 7 Department of Veterinary Pathology, School of Veterinary Medicine, Kitasato University, Towada, Japan; 8 National Institute of Infectious Disease, Shinjuku-ku, Japan; 9 Center for Animal Disease Control, University of Miyazaki, Miyazaki, Japan; 10 Tohoku University Graduate School of Medicine, Sendai, Japan; Liverpool School of Tropical Medicine, UNITED KINGDOM

## Abstract

The direct fluorescent antibody test (dFAT) using brain sample after opening the skull is the standard rabies diagnostic test in animal rabies. However, it is not feasible in many resource-limited settings. Lateral flow devices (LFD) combined with a simple sampling methodology is quicker, simpler, and less hazardous than the standard test and can be a useful tool. We conducted a prospective on-site study to evaluate the diagnostic accuracy of the LFD with the straw sampling method compared with that of the dFAT with the skull opening procedure for post-mortem canine rabies diagnosis. We collected 97 rabies-suspected animals between December 1, 2020 and March 31, 2021. Among the 97 samples, 53 and 50 cases were positive tests for dFAT and LFD, respectively. The sensitivity and specificity of LFD with straw sampling method were 94.3% (95% confidence interval [CI], 84.3–98.8%) and 100% (95% CI, 92.0–100%), respectively. The performance of LFD by the straw sampling method showed relatively high sensitivity and 100% specificity compared with that of dFAT performed on samples collected after opening the skull. This methodology can be beneficial and is a strong tool to overcome limited animal surveillance in remote areas. However, because of our limited sample size, more data using fresh samples on-site and the optimizations are urgently needed for the further implementation in endemic areas.

## Real-world challenge

Limited surveillance for animal rabies often leads to underestimation of its burden in most endemic countries [1]. One of the barriers in most endemic areas is that the standard diagnostic test, namely, the direct fluorescent antibody test (dFAT), can only be performed in a limited number of laboratories because it requires technical expertise for the interpretation of results and complex laboratory equipment such as fluorescence microscopes and incubators. In remote areas far from well-equipped laboratories, the difficulty in obtaining rabies diagnostic results leads to poor surveillance. The standard sampling method to collect brain samples requires the skull to be opened, and this carries a biohazard risk because the procedure can generate aerosols or cause injuries. Considering these factors, skull opening can often only be performed in central laboratories in low- and middle-income countries [2]. A simple sampling methodology using a drinking straw, clamp, or plastic pipette has been recommended as an alternative sampling method by WHO and OIE since the 1980s [2–4]. This sampling procedure is simple and easy and carries a low risk of contamination to the examiner or environment. Therefore, if combined with rapid diagnostic tests, which require minimal equipment, rabies diagnostic tests can be performed in the field or frontline laboratories in remote areas where there are no laboratories equipped with autopsy rooms or microscopes. Direct rapid immunohistochemical tests (DRITs) do not require fluorescent microscopes and have been proven to increase diagnostic capacity in decentralized laboratories [5–7]. Lateral flow devices (LFDs) are quick and easy in comparison with dFAT and DRIT and can be used in areas where neither fluorescent microscopy nor light microscopy is available or practical. Although several LFDs are commercially available, previous studies have reported varied diagnostic accuracies, and inadequate sensitivities have been observed in several LFDs [8–10]. The storage conditions of samples may impact the assessment of LFD, and recent on-site studies have shown high sensitivities and specificities for some LFD [11–14]. However, the lower sensitivity of LFD compared with those of dFAT is still a concern for the implementation of LFD use in the endemic areas [11]. More data and studies using fresh samples on-site are necessary to assess the further implementation of LFD in endemic countries.

LFD combined with straw sampling can be easily performed in the field or resource-limited settings [12]. WHO recommends using brain material from the anatomical region with the highest viral content, such as the brain stem, for LFD [4]. This is supported by several studies that have shown high levels of viral antigens located in the brain stem [15,16]. However, the optimal brain site for sampling cannot be determined when performing the straw sampling or pipette method after dissecting the animal’s head. One question is whether the diagnostic accuracy of LFD performed on the samples obtained by the straw sampling method is comparable to that of the current standard methodology, i.e., dFAT performed on samples collected after opening the skull. To the best of our knowledge, no studies have compared the diagnostic accuracy of LFD using the simplified sampling method with dFAT using samples collected after the skull has been opened. For comparison of the diagnostic accuracy of rapid diagnostics tests and dFAT, most studies have used samples collected by the simplified sampling method for both types of diagnostic test [7,12,14,17]. Mauti and colleagues described the detailed methodology for simplified sampling and LFD [12]. We followed this straw sampling methodology and aimed to gather further data.

## Methods

### Ethics statement

The animal ethical approval was waived by the Institutional Animal Care and Use Committee of the Research Institute of Tropical Medicine in the Philippines. For biosafety clearance, our research protocol was approved by the Biosafety Clearance of RITM (No.190116).

### Sample collection and diagnosis

We conducted a prospective and on-site evaluation study to analyze the performance of LFD using samples collected by the straw sampling method at a regional animal laboratory in Central Luzon (Main Island), which has the highest incidence of animal rabies in the Philippines (S1 STARD Checklist). The detail of methodology has been described previously [11]. This regional laboratory receives the decapitated heads of animals from government agencies or people who find animals with suspected rabies. Any individual can deposit a decapitated head to this laboratory and request rabies testing free of charge. First, we performed straw sampling to collect brain samples as described elsewhere [4,12] (S1 File). We inserted a straw into the foramen magnum with a slight twisting motion toward one of the eyes (S1 File). We used BioMasher II (Nippi, Tokyo, Japan) for brain homogenization, together with an assay buffer of LFD. One staff member performed LFD immediately after sample collection by the straw method. We used Rabies Ag Test Kits (ADTEC, Oita, Japan; lot No. 2011 and 2102) for LFD and followed the manufacturer’s instructions without performing the dilution step (S1 File). Another staff member then opened the skull and collected brain samples from the hippocampus, brain stem, and cerebellum for dFAT. Two independent examiners blindly judged the results without knowing the results of the other test. The sensitivity and specificity of LFD with the straw sampling method were determined and compared with those of dFAT as a reference test (Table 1). We further analyzed those samples for which discrepant results were obtained between LFD with straw sampling and dFAT with sampling from an open skull (Table 2). We performed LFD and real-time reverse transcription polymerase chain reaction (RT-PCR) using brain stem samples collected from the opened skull. The methodology used for RNA extraction and LN34 real-time RT-PCR assay has been described previously [11].

**Table 1:** Sensitivity, specificity, and PPV and NPV of the LFD on samples collected using the straw sampling method compared with those of the dFAT on samples collected after skull opening.

		LFD with straw sampling		Sensitivity (95% CI)	Specificity (95% CI)	PPV (95% CI)	NPV (95% CI)
		Positive	Negative				
**dFAT**	**Positive**	50	3	94.3% (84.3 to 98.8)	100% (92.0 to 100)	100% (92.9 to 100)	93.6% (82.5 to 98.7)
	**Negative**	0	44				

CI, confidence interval; dFAT, direct fluorescent antibody test; LFD, lateral flow device; NPV, negative predictive value; PPV, positive predictive value.

**Table 2:** Results of the 3 discrepant samples.

Sample ID	LFD with straw sampling	dFAT with opened skull sampling	LFD with opened skull sampling	LN34 real-time RT-PCR with opened skull sampling (C_q_ value)
**4032**	Negative	Positive	Positive	Positive (22.54)
**4033**	**Negative**	Positive	Negative	Positive (23.08)
**4051**	Negative	Positive	Positive	Positive (22.39)

C_q_, quantification cycle; dFAT, direct fluorescent antibody test; LFD, lateral flow device; RT-PCR, reverse transcription polymerase chain reaction.

## Results

Between December 1, 2020 and March 31, 2021, 97 samples were submitted from 86 dogs and 11 cats with suspected rabies. The basic characteristics of the submitted animals are shown in S1 Table. A total of 88 (90.7%) samples were either packed on ice or frozen and were well preserved, whereas 9 (9.3%) were unpreserved (S1 Table).

Among the 97 samples, 53 (54.6%) were positive for rabies on dFAT, and 50 were positive on LFD (Table 1). Compared with dFAT, the sensitivity and specificity of LFD using the straw sampling method were 94.3% (95% confidence interval [CI], 84.3% to 98.8%) and 100% (95% CI, 92.0% to 100%), respectively. However, there was a discrepancy between the results obtained by the 2 tests in 3 cases (Table 2). All 3 samples were found to be positive with real-time RT-PCR using the brain stem samples collected after opening the skull, while 2 samples were positive on LFD.

## Discussion

To the best of our knowledge, this is the first report comparing the performance of LFD using a simplified sampling method with that of dFAT performed on samples obtained after opening the skull. Several studies performed LFD and dFAT on samples collected by simplified sampling and showed high performance of LFD [7,12,14,17]. DRIT was also evaluated, and it showed high performance when used for analyzing samples collected by a straw or opening the skull [6]. We showed that the performance of LFD in detecting rabies using brain samples collected by the straw sampling method showed relatively high sensitivity and 100% specificity compared with that of dFAT performed on samples collected after opening the skull. Our results provide additional support for the use of LFD with simplified sampling methods.

However, we detected 3 false-negative samples (3.1%) that were negative for rabies with LFD using the straw sampling method but were positive on dFAT and real-time RT-PCR (Table 2). The discrepancy in one sample (study ID 4033) was possibly due to the low sensitivity of the LFD kit. The other 2 discrepant samples (study ID 4032 and ID 4051) obtained positive results with LFD using open skull sampling; thus, these discrepancies were potentially due to sampling technique error, as the straw sampling method cannot be used to collect samples from a specific part of the brain. However, the major limitation of our study is that we did not perform dFAT or real-time RT-PCR using samples collected by the straw sampling method. In addition, the sample size of this study is limited. Therefore, we were not able to identify the causes of these 3 discrepant results with certainty. Our previous study using brain stem samples collected after opening the skull showed a similar sensitivity of LFD (94%; 95% CI, 87% to 97%) [11]. False-negative results can possibly occur because of not only the lower sensitivities of LFD but also sampling technique error of the straw method. Although the sensitivities of our LFD are relatively high, the false-negative results cannot be ignored because rabies is an almost uniformly fatal infectious disease and a significant public health concern. To overcome the incidence of false-negative results, we currently recommend in our area that head samples should be submitted to the regional central laboratory for a confirmatory test such as dFAT and DRIT with open skull sampling if the only testing in the field or frontline laboratories was performed using LFD with the straw sampling method. There are concerns regarding the false-negative results of LFD with straw sampling. However, this method has substantial advantages over the dFAT opening skull. The positive results can potentially provide benefits such as quick notifications and alerts to the area. Decapitation of the animal’s head is a routine practice in the Philippines when samples are submitted to the central laboratory. Therefore, our methodology can be easily adopted in many settings and is a suitable option for on-site diagnosis in remote settings.

Our study has other limitations. Cutoff heads were removed by the owner or local governments. Therefore, the decapitation procedure was not standardized. We observed that some heads were not removed before the first cervical vertebra. The long distance to the brain stem might affect the collection of brain samples and sensitivity of the test. We did not validate the performance of the proposed methodology under field conditions. Further studies should evaluate the diagnostic performance, feasibility, and safety of performing on-site LFD. All samples in our study were fresh during testing, and we assumed that the results were not affected by sample transportation.

This methodology can be beneficial and is a strong tool to overcome limited animal surveillance in remote areas. However, because of the limited data in our study, more data and optimization are urgently needed for further implementation.

**Key Learning Points**

- Lateral flow device (LFD) combined with the straw sampling method can potentially provide great opportunities to diagnose animal rabies in resource-limited settings.
- A potential concern of LFD testing is low sensitivity; sensitivities vary between kits.
- Our method of LFD combined with straw sampling showed relatively high sensitivity and 100% specificity; however, there are still concerns regarding false-negative results.
- For further implementation of LFD with the straw sampling method in the field or laboratories in remote areas, more on-site data using fresh samples are needed to identify the most appropriate methodology.

## Supporting information

S1 STARD Checklist STARD checklist and the diagram. STARD, STAndards for the Reporting of Diagnostic accuracy studies.
(DOCX)

S1 File Operation manual for the straw sampling method and LFDs. LFD, lateral flow device.
(DOCX)

S1 Table Characteristics of animals with suspected rabies and the results of dFAT. dFAT, direct fluorescent antibody test.
(PDF)
