# Supplementary material for: Lateral flow devices for samples collected by straw sampling method for postmortem canine rabies diagnosis
Source: PLoS Negl Trop Dis. 2021 Dec 9;15(12):e0009891. doi: 10.1371/journal.pntd.0009891 (PMC8659307; doi:10.1371/journal.pntd.0009891)
Supplement: S1 File — LFD, lateral flow device. (DOCX) [file pntd.0009891.s002.docx]

**Supporting information 1.**

**Operation manual of straw sampling method and**

**lateral flow devices (LFD)**

**I. Materials Needed for straw sampling**

Scalpel, Scalpel blade, Scissors, Forceps

Rabies Ag test kits (including Test card, Assay Buffer, Sample masher kit (1,5ml tube and pestle), Dropper, Plastic straw, Wooden applicator stick)

**II. Procedure**

Step 1. Prepare all necessary materials

Step 2. Transfer assay buffer to 1.5ml tube (until middle line)


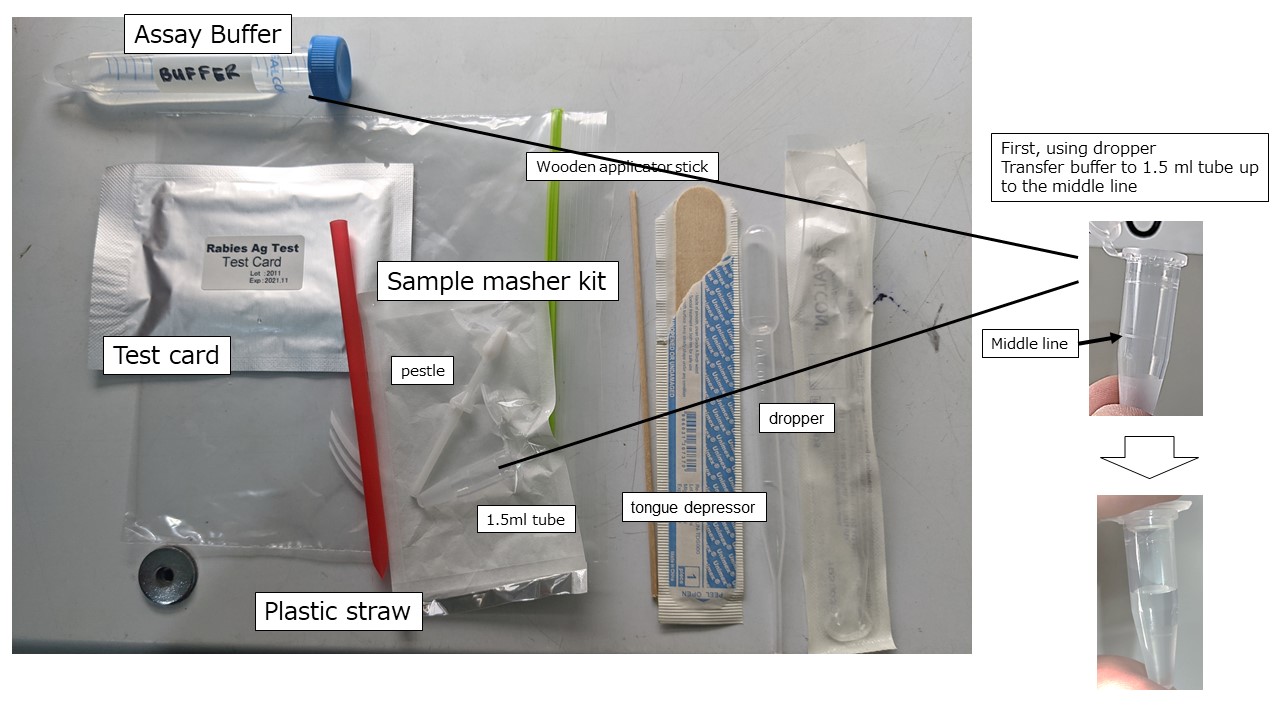


Step 3. Wear the appropriate PPE (Personal Protective Equipment)

Step 4. Fix the animal's head so as not to get injured during the work.

Step 5. Remove the muscle around the spinal cord and expose the foramen magnum (Fig 1).

Step 6. (To take brain sample) Insert the straw [1] into the foramen magnum with a slight twisting movement towards one of the eyes (Fig 2). This will allow samples to be taken from the medulla oblongata, the base of the cerebellum, the hippocampus (Ammon’s horn) and the cerebral cortex (Fig 3).

Before withdrawing the straw, pinch it between the fingers in order to prevent brain tissues from escaping in the straw.

| Fig 1.  Exposure of the external occipital protuberance | Fig 2.  Insert the straw [1] into the occipital foramen with a slight twisting movement towards one of the eyes (Step 6) |
| --- | --- |
| 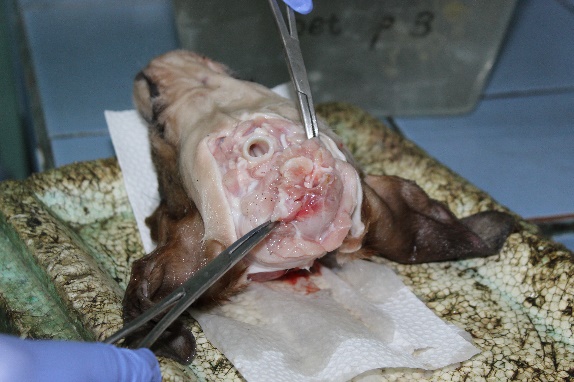 | 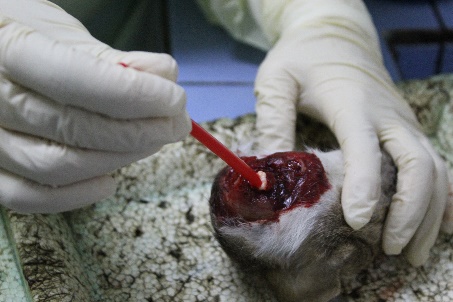 |
| Fig 3.  Sagittal section demonstrating the insertion of the straw (Step 6) |  |
| 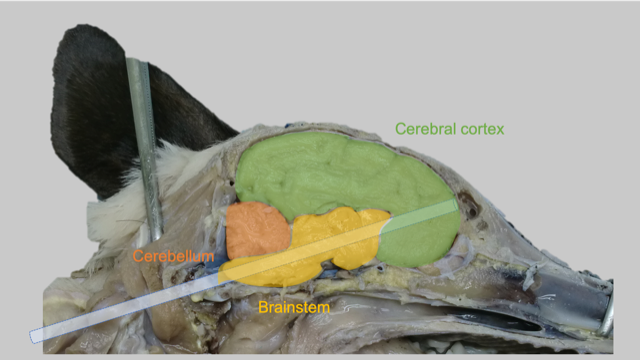 |  |

| 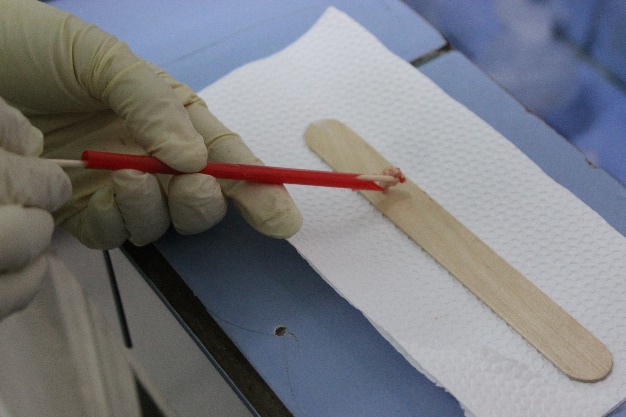Fig 4. Extract the content into a tongue depressor or container. | Step 7.  After withdrawing the straw, extract the content by pushing a wooden applicator stick in the straw to a tongue depressor or container (Fig 4). |
| --- | --- |
| 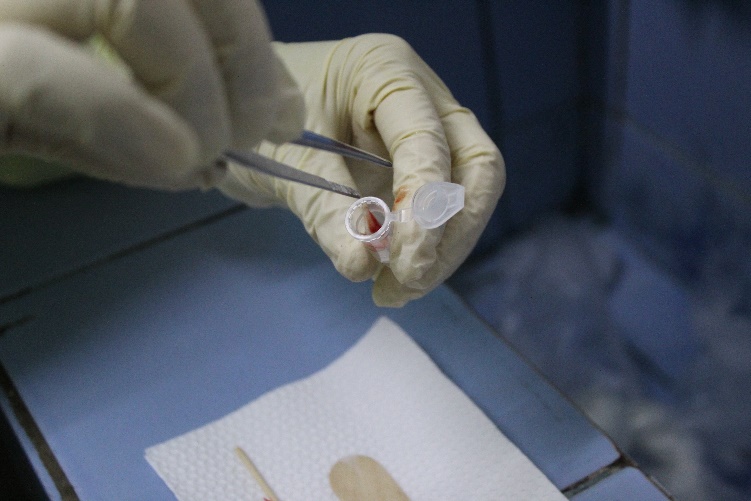Fig 5-1. Transfer the brain sample into 1.5ml tube. | Step 8.  Using plain forceps, transfer small size (about 5mm*5mm, Fig 5-2) of the brain sample to a 1.5ml tube filled with Assay Buffer (Fig 5-1).  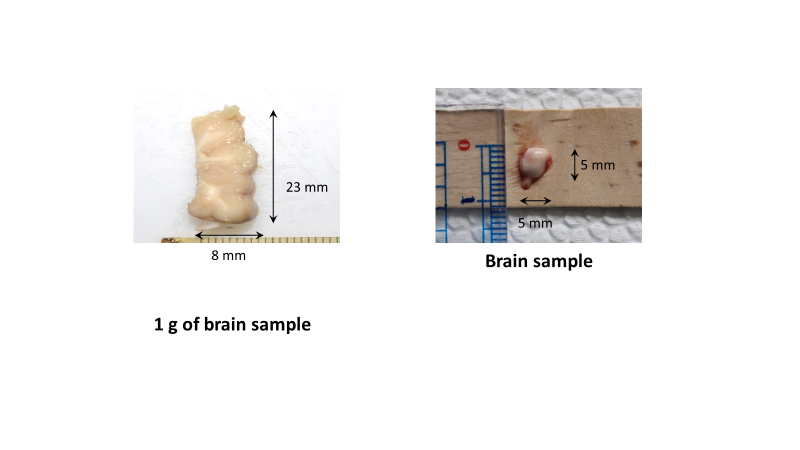  Fig 5-2 Size of Brain sample |
| 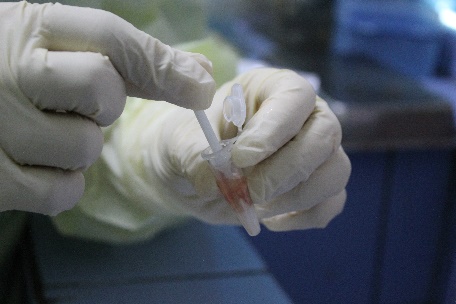Fig 6. Homogenizing brain sample using Masher. | Step 9.  Homogenize completely using the pestle (sample masher) (Figure 6). |
| 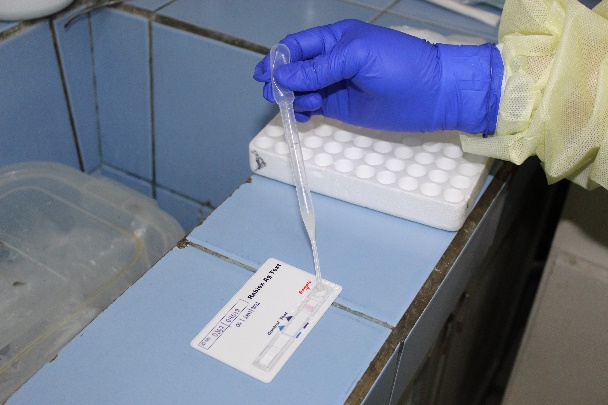Fig 7. Applying 3 drops of homogenized sample to test card. | Step 10.  Using a dropper (or Pipette), apply 3 drops (100uL) of the homogenized sample to the test card (Fig 7). |

|   Fig 8. The images and the interpretations of LFD | Step 11.  Read result after 10-15 min. Interpretation: Control line only is a negative result. Appearance of both test line and control line is a positive result. Appearance of test line only is invalid; no appearance of both lines is also invalid  (Fig 8). |
| --- | --- |

Reference

1. Mauti S, Léchenne M, Naïssengar S, Traoré A, Kallo V, Kouakou C, et al. Field Postmortem Rabies Rapid Immunochromatographic Diagnostic Test for Resource-Limited Settings with Further Molecular Applications. JoVE. 2020; 60008. doi:10.3791/60008
