# Supplementary material for: Lateral flow devices for samples collected by straw sampling method for postmortem canine rabies diagnosis
Source: PLoS Negl Trop Dis. 2021 Dec 9;15(12):e0009891. doi: 10.1371/journal.pntd.0009891 (PMC8659307; doi:10.1371/journal.pntd.0009891)
Supplement: S1 Table — dFAT, direct fluorescent antibody test. (PDF) [file pntd.0009891.s003.pdf]

Supporting information

S1 Table. Characteristics of rabies-suspected animals and the results of direct fluorescent antibody test (dFAT)

Category		Total (%)	dFAT Negative (%)	dFAT Positive (%)
Province where the submitted samples were found	Near the RADDL III (PAMPANGA)	57 (58.8)	28 (49.1)	29 (50.9)
	Far from RADDL III (Outside PAMPANGA)	40 (41.2)	16 (40.0)	24 (60.0)
Species	Dog	86 (88.7)	33 (38.4)	53 (61.3)
	Cat	11 (11.3)	11 (100)	0 (0)
Age (n=77)	< 1 year	49 (63.6)	28 (57.1)	21 (53.9)
	1 year	7 (9.1)	2 (28.6)	5 (71.4)
	2 years	5 (6.5)	1 (20.0)	4 (80.0)
	> 2 years	16 (20.8)	7 (43.8)	9 (56.3)
Sex (n=85)	Female	34 (40.0)	19 (55.9)	15 (44.1)
	Male	51 (60.0)	20 (39.2)	31 (60.8)
Body size	Small (<10kg)	75 (77.3)	40 (53.3)	35 (46.7)
	Middle or Large (≥10kg)	22 (22.7)	4 (9.1)	18 (81.8)
Storage condition during and prior to the delivery	Unpreserved *	9 (9.3)	7 (77.8)	2 (22.2)
	Well preserved	88 (90.7)	37 (42.1)	51 (58.0)

* Stored more than 3 hours at room temperature

RADDL, Regional animal disease diagnostic laboratory; dFAT, direct fluorescent antibody test
